# Supplementary material for: Enhancing brain age estimation with structural MRI and synthesized cerebral blood volume maps
Source: Brain Commun. 2026 Jul 22;8(5):fcag283. doi: 10.1093/braincomms/fcag283 (PMC13532576; doi:10.1093/braincomms/fcag283)

3D GradCAM Visualization of Top 20% Gradient Values Averaged for Cognitively Normal Participants from the test set of ages 65-100 from the MRI and DeepCBV CNNs to qualitatively characterize the different regions each modality emphasizes

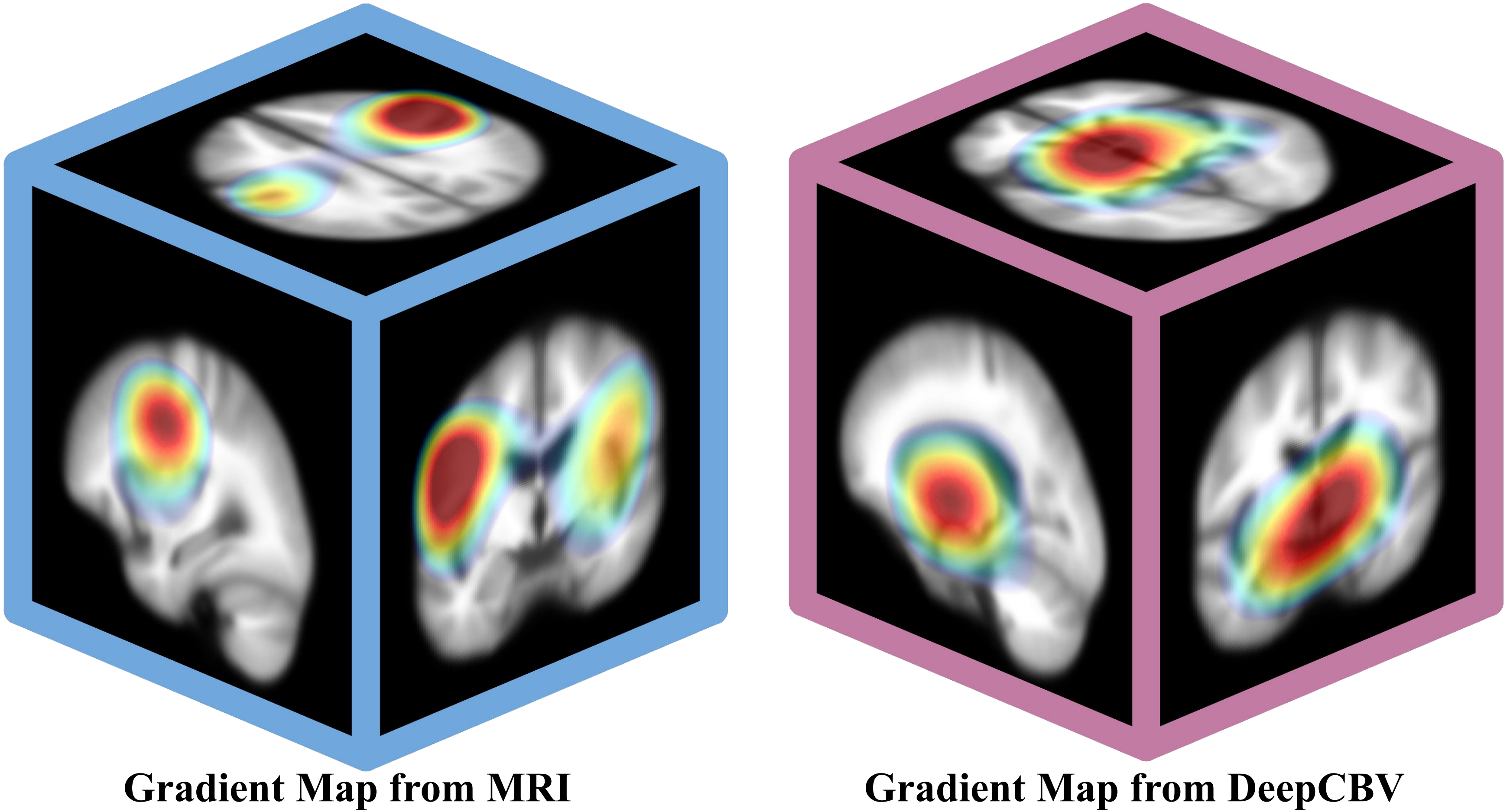

Supplement: Supplementary file 1 [file fcag283_supplementary_data.pdf]
